# Sternal Resections: An Attempt to Find the Ideal Reconstruction Method

**DOI:** 10.3390/medicina61040763

**Published:** 2025-04-21

**Authors:** Emanuel Palade, Ioana-Medeea Titu, Lucian Fodor, Ion Mircea Ciorba, Ion Jentimir, Florin Teterea, Monica Mlesnite, Ioana Tichil

**Affiliations:** 1 Department of Surgery, Iuliu Hatieganu University of Medicine and Pharmacy, 400000 Cluj-Napoca, Romania; emanuel.palade@umfcluj.ro; 2 Thoracic Surgery Clinic, Leon Daniello Clinical Hospital of Pneumology, 400371 Cluj-Napoca, Romania; 3 Interservisan Medical and Surgical Center, 400431 Cluj-Napoca, Romania; 4 Department 1—Morpho-Functional Sciences, Iuliu Hatieganu University of Medicine and Pharmacy, 400006 Cluj-Napoca, Romania

**Keywords:** sternectomy, chest wall reconstruction, spider-web technique, anterolateral thigh flap, soft tissue reconstruction

## Abstract

*Background and Objectives:* Sternal resections, although rare, pose significant challenges for the reconstruction of large anterior chest wall defects. Both stability and soft tissue reconstruction are essential for preventing respiratory complications and ensure structural stability. Despite the variety of techniques proposed, no consensus exists on an optimal method. Herein, we present our institutional experience using the novel “spider-web” technique combined with an anterolateral thigh (ALT) free flap for chest wall and soft tissue reconstruction following extensive sternectomies. *Materials and Methods:* Between January 2023 and November 2024, five female patients underwent partial or total sternectomy for oncologic indications. Chest wall stability was restored using the “spider-web” technique–based on non-resorbable polyester threads arranged in a geometric web pattern reinforced with polypropylene mesh–followed by soft tissue reconstruction using a free ALT myocutaneous flap. Data on demographics, surgical details, postoperative outcomes, and complications were prospectively analyzed. *Results:* Resections included two partial and three total sternectomies. The mean operative time was 385 min (range: 330–435 min). All patients were extubated postoperatively without the need for respiratory support. The “spider-web” construct provided adequate chest wall stability with no cases of paradoxical movement or chronic pain. No flap loss occurred; one case required revision for venous thrombosis, and one hematoma at the donor site was evacuated. No infections or wound dehiscence were observed. The median hospital stay was 11 days (SD ± 1.67 days), and 30-day mortality was 0%. Median follow-up was 10 months (SD ± 6.55 months), without long-term complications. *Conclusions:* The “spider-web” technique, combined with ALT myocutaneous free flap, is a simple, reliable, and cost-effective method for the reconstructing extensive chest wall defects following sternectomy. Its technical versatility and favorable outcomes suggest it as a valuable option, offering both structural stability and soft tissue coverage with minimal morbidity.

## 1. Introduction

Partial or total sternal resections (sternectomy) are seldom performed operations, usually resulting in a large chest wall defect with a high risk of severe complications if not appropriately reconstructed. Studies analyzing large series of chest wall resections across various indications report an incidence of about 8% for sternectomies [1]. In absolute numbers, in single-center studies, patients’ cohorts range from 14 to 60 patients [2,3]. For total sternectomy, the caseload in these studies is significantly lower (three to six patients), with only one study (Ahmad U et al., 2016) reporting on 13 such procedures [3,4,5].

The majority of sternal resections are performed for neoplastic diseases. Benign sternal tumors (chondroma, bone cyst, fibrous dysplasia, etc.) are exceedingly rare, while primary sternal tumors that are malignant (chondrosarcoma followed by osteosarcoma) constitute most cases [6]. Contrary to other chest wall locations, sternal resections for secondary tumors account for up to 33% of all sternectomies [3]. Among sternal metastases, 40–50% originate from breast cancer, followed by papillary thyroid carcinoma and renal cancer [3,6]. Less common indications for sternectomy include sternal infections, traumatic injuries, and radionecrosis [6,7].

Chest wall defects after sternectomy, similar to other anterior and anterolateral defects, have a more profound negative impact on pulmonary function compared with lateral and posterior defects due to paradoxical respiratory movements. Therefore, stable chest wall reconstruction is mandatory for defects larger than 5 cm in diameter [8,9]. Large defects are common in this location, as a macroscopic resection margin of at least 3 cm is recommended for malignant lesions to minimize recurrence risk [10]. Depending on the resection extent (partial or total sternectomy) and the preservation or not of the manubrium sterni, several techniques and materials can be used for reconstruction. Table 1 summarizes the different materials, their benefits and drawbacks, recent developments, and future perspectives [6,8,9,11].

In all cases, the restoration of the chest wall stability must also include soft tissue reconstruction [13]. The primary objectives of soft tissue reconstruction include protecting the prosthetic material from infection, covering the resulting defect, and enhancing chest wall stability while safeguarding underlying organs [4]. Choosing the right technique for soft tissue reconstruction depends on the defect size and availability and quality of the surrounding tissue. Although it is easier to use local or regional flaps for large-size defects, microsurgical reconstruction with free flaps seems to be the best option. Good tissue coverage is one of the important steps for successful treatment. Muscle composite free flaps offer one of the best options for reconstruction. For malignant sternal tumors, large soft tissue defects involving the skin are common. A consensus study highlighted that up to 41% of respondents supported the need for extended resection, including skin overlying the tumor, even in the absence of direct invasion on imaging or palpation [10]. Consequently, myocutaneous flaps are frequently used for increasing surgical complexity, operative time, and the risk of complications. Therefore, the involvement of plastic surgeons in the management of such cases plays a key role [13]. Unlike chest wall reconstruction, where a large variety of synthetic materials or allogenic transplants can be used, soft tissue reconstruction relies exclusively on autologous tissue. Table 2 outlines the different soft tissue reconstruction methods available for covering sternal defects [9,13,14].

In our institution, regardless of their location, the majority of chest wall defects are reconstructed using the “spider-web” technique. We consider this approach advantageous due to its technical simplicity, applicability to defects of varying sizes and locations, cost-effectiveness, and ability to provide adequate stability using readily available materials.

Additionally, our standard approach involves the routine use of the ALT myocutaneous flap for reconstruction after total or extensive partial sternectomies. This flap is favored due to its reliable vascular pedicle, capacity to cover large soft tissue defects—including a significant skin paddle, compatibility with a simultaneous two-team approach, and minimal donor site morbidity.

The rarity and complexity of sternal reconstructions underline the need for a multidisciplinary approach (thoracic surgery, plastic surgery, medical oncology, and radiation oncology) in experienced centers to ensure optimal patient outcomes [13].

## 2. Materials and Methods

Between January 2023 and November 2024, five sternectomies followed by chest wall and soft tissue reconstruction were performed in our department. The extent of sternal resections was classified according to our internal classification system (see Table 3). Parameters regarding demographics of the analyzed group, diagnosis, tumor size, type of chest wall and soft tissue reconstruction, duration of surgery, postoperative length of stay (LOS), 30-day morbidity, and 30-day mortality were prospectively assessed.

### 2.1. “Spider Web” Reconstruction Technique Following Sternectomy

All resections were performed for neoplastic lesions, necessitating resection with tumor-free margins. For oncological safety reasons, the excision extended laterally to include adjacent costal cartilages and, in some cases, the anterior portions of the ribs. This resulted in large anterior or anterolateral chest wall defects in every case. The primary stabilization layer consisted of a web-like structure (“spider-web”) constructed using strong, non-resorbable polyester threads (size 1 or 2). The web-like construction begins with the placement of two longitudinal threads approximately 1–2 cm lateral to the remaining rib edges. To minimize pressure on the intercostal nerves, these two threads are placed in a continuous figure-eight fashion at the cranial margins of the ribs. Transversal threads are then placed at the level of each intercostal space, which is anchored around the longitudinal threads. To reduce postoperative pain, trans- or pericostal wire placement was generally avoided. One or two additional longitudinal threads may be placed within the defect for further stabilization. All threads must be securely tied, ensuring they are not overstretched. This generally leads to a size reduction of the chest wall defect, but care must be taken to avoid excessive approximation of the resected rib ends, which could contribute to postoperative pain. Each thread crossing is additionally secured with separate ligatures to ensure that tension is distributed in all directions. In cases with resection of manubrium sterni, clavicular fixation was performed using one of two techniques, depending on the extent of resection. If no or only limited resection of one or both clavicles is performed, a strong, non-resorbable wire is used to secure the clavicle to the corresponding first rib. However, if the clavicle resection extends more laterally, this technique is not feasible due to the risk of compressing the subclavian vessels. In such cases, a No. 5 steel wire is placed between the clavicle ends for fixation. To prevent tracheal compression, the steel wire is positioned in a figure-eight configuration.

**Figure 1:**
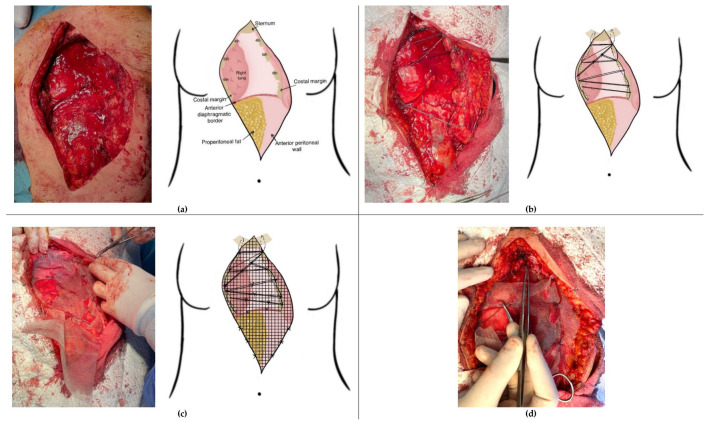
Surgical steps of the “spider web” reconstruction technique following sternectomy: (**a**) resection with tumor-free margins; (**b**) creation of a “spider-web” stabilization layer using durable, non-resorbable polyester threads; (**c**) placement of a second reinforcement layer with polypropylene mesh; and (**d**) leaving the superior aspect of the defect uncovered to allow vascular anastomosis of the ALT flap.

Following the “spider-web” stabilization, a polypropylene mesh was applied as a second reinforcement layer. The mesh is secured under moderate tension using interrupted non-resorbable sutures along its margins and at several intersections of the “spider-web”. A window in the mesh was prepared in the upper part to facilitate access for vessel anastomosis to the recipient site. Figure 1 highlights the procedural steps of the “spider-web” technique.

Simultaneous soft tissue reconstruction was performed using a free ALT composite flap in all cases. The ALT flap was first described in 1984 by Wong et al. initially as a septocutaneous perforator flap [19]. Since then, our understanding of perforator anatomy has expanded, making the ALT flap today a workhouse flap for head and neck, upper and lower limb and trunk reconstructions. Blood supply to the flap is provided by perforators, mainly from the descending branches of the lateral circumflex femoral artery. Vascular anatomy classification includes five types, as described in Table 4 [20].

For all five patients we used type IV ALT flaps based on multiple perforators from the descending branch of the lateral circumflex femoral artery. All ALT flaps were designed to include along with the skin island vastus lateralis muscle mass generally with preservation of the proximal perforator for both soft tissue coverage and dead space closure.

Preoperative mapping of perforators was performed using a hand-held Doppler device and provided accurate perforator identification as correlated with surgical findings. No significant difference in perforator number and distribution was observed regarding the dominant and non-dominant sides. This consists of previously reported data [21]. The flap harvesting was used to include fascia lata and part of the rectus femoris muscle. Careful dissection was performed to preserve the motor branches of the femoral nerve. The pedicle dissection war continued proximally to the descending branch of the circumflex femoral vessels. I.V. 3.000 Units of heparin were given five minutes before flap detachment. The recipient vessels were the internal mammary artery and vein in four cases and the left brachiocephalic vein in a single case. The arterial anastomosis was performed end to end with Prolene 8–0 suture in all cases. The vein was sutured end to end to the internal mammary in four cases and end to side to the brachiocephalic vein due to the lack of internal mammary vessels. The fascia lata was interposed between the pedicle and mesh to avoid possible adhesions and vascular thrombosis.

All donor sites were primarily closed, and one patient developed a hematoma that was surgically evacuated.

**Figure 2:**
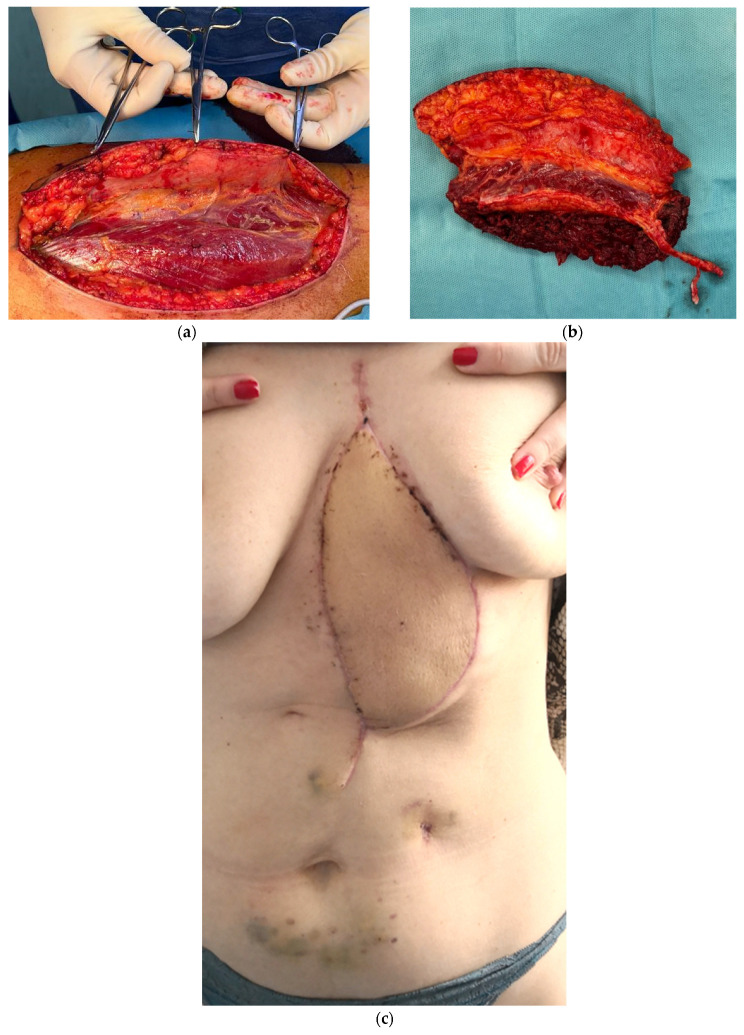
Soft tissue reconstruction after extended total sternectomy: (**a**,**b**) myocutaneous ALT flap harvested from left thigh; (**c**) postoperative aspect at 1-month follow-up.

Postop monitoring of flap viability was performed in the ICU through clinical and Doppler hourly assessment within the first 24 h and 3 hourly on the ward within the next 48 h. Patients received anticoagulation with Clexane 4000 UI/day for the admission period and 75 mg of Aspirin for a 3-week period. One patient presented venous thrombosis of the flap pedicle within the first 24 h post-surgery, which led to surgical exploration, trombectomy and reanastomosis that promptly prevented flap loss. No other flap-related immediate or long-term complications were recorded. Figure 2 showcases the ALT free flap harvesting and the 1-month postoperative result.

### 2.2. Statistical Analysis

For descriptive statistics, mean values with ranges and medians with standard deviations were calculated. Our findings were compared with data from the literature. For this purpose, a review of publications on sternectomy over the past 10 years was conducted, which yielded 58 articles. From this initial pool, we selected 12 articles that met the following inclusion criteria: full-text availability, publication in English, and relevance to oncological pathology. The exclusion criteria comprised studies focusing on non-oncological pathology, partial sternectomy, case reports, letters to the editor, review articles, unavailability of full text, and publications in languages other than English. Additionally, a secondary screening of the reference lists of the selected articles identified 10 additional relevant studies, bringing the final number of reviewed articles to 22.

## 3. Results

Between January 2023 and November 2024, five female patients underwent sternectomy (type B in two cases, type C in three cases). Four resections were performed for malignant lesions (two breast cancer metastasis, one thyroid cancer metastasis, and one Hodgkin lymphoma), while one was indicated for recurrence of previously incompletely resected desmoid fibromatosis. Chest wall stability was reconstructed using the “spider-web” technique combined with polypropylene mesh in four cases, whereas one patient received titanium bars (STRATOS™, MedXpert GmbH, Eschbach, Germany) covered with polypropylene mesh. In all patients, soft tissue reconstruction was achieved using an ALT myocutaneous free flap.

All procedures were performed by two surgical teams–thoracic surgeons for chest wall resection and stabilization and plastic surgeons for soft tissue reconstruction. To reduce operative time, both teams worked simultaneously. The mean duration of surgery was 385 min (range: 330–435 min). All patients were extubated immediately postoperatively and did not require mechanical respiratory support over the entire postoperative course.

### 3.1. Postoperative Course and Complications

No complications related to the chest wall reconstruction, including paradoxical respiratory movement, infection, significant acute pain, or chronic pain, were encountered. In one case, venous thrombosis of the vascular flap pedicle was suspected approximately 24 h postoperatively. Emergency surgical exploration, vein desobstruction, and reanastomosis were performed successfully, with no further complications. In another case, a hematoma at the flap donor site required surgical evacuation with no subsequent complications. One patient developed mild pneumonia but no respiratory insufficiency or need for additional respiratory support. Among the five patients included in this study, three (60%) required a postoperative blood transfusion. Out of the five patients included in this study, three required postoperative blood transfusion. Each of these patients received a single unit of packed red blood cells, indicating that no major intraoperative postoperative blood loss occurred. No flap failure occurred, and 30-day mortality was 0%. The LOS varied among the patients. The median LOS was 11 days. The range of hospitalization spanned from 9 to 14 days, with a standard deviation (SD) of ±1.67 days.

The median follow-up period was 10 months, with an SD of ±6.55 months.

The demographic and surgical characteristics of the patient cohort are summarized in Table 5.

### 3.2. Case-Specific Surgical Considerations

In three cases, specific surgical considerations warrant discussion. In the case of recurrent desmoid fibromatosis (incomplete mass removal without chest wall resection performed in another institution), complete tumor excision was achieved by resecting the sternal body (type B resection), the left anterior chest wall beginning with the fourth rib, and extending laterally to the midclavicular line and the upper portion of the rectus abdominis muscle, including the properitoneal adipose tissue up to the left semilunar line. The overlying skin was resected en bloc with the tumor. This very large defect was reconstructed using a “spider-web” technique covered by polypropylene mesh for the chest wall and an additional polypropylene mesh for the abdominal wall. For the soft tissue reconstruction, an ALT myocutaneous flap harvested from the right thigh was used. A venous thrombosis of the flap’s vascular pedicle was suspected 24 h postoperatively, necessitating revision of the venous anastomosis, which was successfully performed with no further complications.

The patient with sternal Hodgkin lymphoma presented a necrotic mass with skin ulceration at the level of the sternal body. Histological diagnosis and treatment were hampered by the superinfection of the tumor. Despite chemotherapy, radiotherapy, and immunotherapy, a residual sternal mass and a cutaneous fistula persisted. A total sternectomy (type C sternal resection), including the affected skin, was performed. In this case, we chose to use titanium bars (STRATOS™ system) covered with polypropylene mesh for stability, and soft tissue reconstruction was completed with an ALT myocutaneous flap. The postoperative course was uneventful until the clinical and radiological follow-up at 7 months revealed a dislocation of all three titanium bars, although the patient remained asymptomatic. As all bars are fixed with non-resorbable wires to the polypropylene mesh and the tip of the bars displaced anteriorly (in front of the left anterior chest wall), a decision to not explant the bars was made.

In the third case, the patient developed a sternal recurrence of a poorly differentiated thyroid carcinoma approximately two years after the initial resection performed in another institution. The first surgical treatment included resection of the manubrium sterni, right sternoclavicular joint, and first right chondrosternal joint, followed by adjuvant radiotherapy. The residual sternum was resected en bloc with soft tissue and skin scar. The chest wall stability was obtained using the “spider-web” technique, and the soft tissues were reconstructed using an ALT myocutaneous flap. Given the patient’s prior resection and radiotherapy, the internal mammary veins were unsuitable for anastomosis. Instead, venous drainage of the flap was redirected to the left brachiocephalic vein. Except for a hematoma at the donor site, which was evacuated, no further complications occurred.

## 4. Discussion

Sternectomies (partial or total) are rare surgical procedures primarily performed for oncologic indications. The existing literature on this topic is relatively scarce, offering insights into the multitude of techniques described for sternal reconstruction (see Table 1 and Table 2); however, it lacks sufficient scientific evidence to establish a standard procedure [6,11,22]. Two key surgical considerations are pivotal in reconstructions after sternectomies: first, large anterior chest wall defects pose a greater risk of severe respiratory impairment compared to defects in other thoracic regions, and second, soft tissue reconstruction frequently necessitates complex plastic surgical procedures [8,9,11,13].

As the extent of sternal resection has an impact on the reconstruction technique and possible postoperative morbidities—paradoxical movements, respiratory impairment, clavicular displacement, and shoulder dysfunction—it appears to be of practical importance to classify the type of resection. A first, generally accepted classification is in partial and total resections. In 2012, Fabre D. et al. proposed a classification based on the longitudinal extent of the resection, categorizing it as partial (<90% of sternum), subtotal (≥90% but <100%) and total (100%) resection [23]. Aranda J.L. et al. went further, proposing two algorithms, one to orient the extent of the resection and the other one to choose the type of reconstruction. Thus, the sternum is divided into three parts: manubrium, middle sternal body, and lower sternal body plus xiphoid. In cases with tumor involvement of only one-part, partial sternectomy and reconstruction using flexible (mesh) materials can be performed. If the tumor extends to more than one part, subtotal or total resection followed by reconstruction using rigid materials is recommended by the authors [6].

In our study, we proposed a classification of sternal resections in three types (Table 3), primarily based on considerations related to the stability of the reconstruction. For resections involving the manubrium sterni (type A resections), clavicular fixation is mandatory to maintain shoulder girdle stability, as for resections involving the corpus sterni (type B resections) the chest wall stability must be reinsured. Finally, in total sternal resections (type C), both clavicular fixation and chest wall stabilization must be achieved.

From the multitude of materials that can be used for chest wall reconstruction following sternectomy, the majority of surgeons (82.5% in a consensus study from Wang L et al.) prefer meshes, as these materials are widely available at reduced costs, easy to handle and store, adaptable to various defect sizes and shapes, and offer satisfactory stability and mediastinal organ protection when covered with muscle flaps [6,8,10,11]. This seems to be confirmed by two recent studies on sternal oncologic resections. Banuelos J. et al. (2019) investigated 60 patients with different extents of sternectomy, with 76.7% receiving polytetrafluoroethylene (PTFE) mesh-based reconstruction [3]. Similarly, de Macedo J.P.C. et al. (2024) reported on 45 sternectomies (22 partial, 18 subtotal, 5 total) reconstructed with flexible material (polypropylene mesh) in 86.6% of cases [24]. Interestingly, despite the rate of respiratory complications being low in both studies, such complications were associated with high mortality among affected patients. In the study by Banuelos J. et al., only one patient developed postoperative pneumonia (1.7% rate) and died because of respiratory failure [3]. In the de Macedo J.P.C. et al. study, two out of three patients with respiratory insufficiency (about 7% complication rate) succumbed consequently [24]. Local complications (seroma, infection, flap venous congestion, or partial necrosis) had a relatively high incidence (23.3% and 33.3%, respectively), with surgical site infections being encountered in 18% (11 patients, 9 requiring additional surgery) and 11% (five patients, three requiring mesh removal). The mortality rate in both studies ranged from 0% to 9.5%, aligning with studies published by other authors (30-day mortality of 1.6% in the study by Banuelos J et al. and 6% in-hospital mortality in the study by de Macedo JCP et al.) [3,24]. Similar results were reported by Colella S et al. in their recently published systematic review (16 studies, with a total of 197 partial or total sternectomies out of 1.089 patients) analyzing chest wall reconstructions using non-rigid materials. With an average mortality of 1.04% (ranging from 0% to 5.4%) and an average infection rate of 3.8% (ranging from 2% to 17.3%), both parameters were low, suggesting a possible advantage for the use of these materials (synthetic or biologic meshes) [22].

In an attempt to reduce the rate of respiratory complications, several materials and techniques for rigid reconstruction have been developed (see Table 1). The first widely adopted material was methyl methacrylate, often used in combination with two layers of polypropylene mesh (“sandwich” technique). A multitude of other techniques emerged over time, including titanium bares (e.g., STRATOS™ system) and cadaveric cryopreserved sternal allograft (sternal transplantation) as the most notable developments. All these methods are relatively easy to use, provide completely stable reconstruction and offer very good cosmetic results, but they are associated with specific disadvantages and possible complications. Excepting cadaveric sternal allografts, both methyl methacrylate and titanium bares have a significant risk of infection, can be subjected to fracture or displacement of the material, and are associated with increased pain due to the lack of flexibility. For methyl methacrylate, wound complications are reported in 10–20% of cases, with about 5% requiring removal. For titanium bars, fracture and displacement can reach an 11% rate [11].

Regarding the protection from respiratory complications as a result of better chest wall stability, studies analyzing this outcome based on a direct comparison between non-rigid and rigid reconstruction methods for sternectomies are limited. Spicer J.D. et al. (2016) [1] retrospectively analyzed 427 chest wall resections, including 33 sternectomies. The reported respiratory complications included pneumonia, reintubation, aspiration, pleural effusion, pneumothorax, and pulmonary embolism. The overall pulmonary complication rate was high (24%) and was influenced by resection extension (number of ribs) and concomitant pulmonary resection (lobectomy). However, severe respiratory complications requiring reintubation were rare (about 3%), with no significant difference between rigid (two out of 82 patients, 2.4%) and flexible (11 out of 345 patients, 3.2%) reconstructions. Although a subgroup analysis on sternal resections was not performed, the difference between rigid and flexible reconstruction regarding respiratory complications was not significant (*p* = 0.401). Interestingly, the local infectious complications were rare (13 cases, 3%), not being influenced by the material used (permanent or absorbable). The 30-day mortality rate was 1%, which is a very low rate for the complex surgery performed [1].

First introduced by Marulli G. et al. (2010), cadaveric cryopreserved sternal allografts are considered a viable alternative to other rigid reconstruction methods, especially for sternal infection and dehiscence after sternotomy [25]. Several subsequent publications (Marulli G. et al., 2017; Marulli G. et al., 2020) have documented the group’s expanding positive experience with this technique [12,26]. Probably the largest series, including 58 sternectomies (30 total and 28 subtotal) reconstructed with cadaveric allograft from 7 centers, was published by Dell’Amore A et al. (2021) [7]. In contrast to other studies on sternectomies where primary or secondary tumors are the main indication, in this study, the proportion of non-neoplastic indications was 52%. Among these cases, 83% (n = 25) involved post-sternotomy dehiscence, highlighting this condition as a major indication for this technique due to the resistance of the material to infection. Titanium bars were used to stabilize the allograft (median: 4 bars; interquartile range [IQR]: 3–4), and soft tissue coverage was performed in 89% of cases (different types of muscle flaps in 50 and omentum transposition in two cases). The median operative time was 154 min (range: 117–429 min), the median intensive care unit stay was 3 days (range: 1–36 days), and the median ventilation duration was 8 days (range: 1–312 days). With a median hospital stay of 11 days ([IQR]: 8–18), a low rate of respiratory complications (7%) and wound dehiscence (7%), an acceptable 30-day mortality of 5% and no complications related to the bone-allograft, the results are encouraging [7]. Although the method seems to have clear advantages over other reconstruction methods (see Table 1), the worldwide use of this technique remains very limited. This is probably related to difficulties in procurement and storage (tissue bank) of the allograft or to regulatory constraints.

The “spider web” technique was developed to combine the benefits of existing reconstruction methods while minimizing their drawbacks. In our institution, this technique has been widely used to reconstruct all types of chest wall defects, regardless of their location, shape, or extent. In our experience, the method provides stable reconstruction and proves to be simple, cost-effective, and universally applicable. The materials used are largely available, translucent to X-rays, and inert to body fluids. While its only theoretical drawback is reduced mediastinal organ protection, this is mitigated by routine musculocutaneous flap coverage.

Similar to other studies on sternectomies, the number of extended sternal resections and reconstructions in our series is small, as this type of surgery is rarely performed. In the largest published series on sternectomy, the number of total sternal resections generally ranges from three to six patients [3,4]. In our study, all patients were extubated immediately after surgery and remained off mechanical ventilation throughout the postoperative course. All reconstructions were stable, with no complications related to the “spider web” technique. The only case of postoperative pneumonia was likely unrelated to the reconstruction itself, did not lead to respiratory insufficiency (manifesting only as a radiologic pulmonary infiltrate), and was easily managed with antibiotic therapy and respiratory physiotherapy. Conversely, in the single case where titanium bars were utilized, all three bars experienced late displacement. While displacement of titanium bars has been reported in approximately 11% of cases, some studies indicate fracture and displacement rates as high as 44.8% on follow-up CT scans [27]. Additionally, the risk of failure for titanium implants is higher in anterior chest wall reconstructions, particularly when more than two bars are used [27]. Although only 10.3% of affected patients develop symptoms [27], and no further complications occurred in our case, this experience discourages the future use of titanium bars for extended chest wall reconstructions in our practice.

A key distinction between our study and other published reports on sternectomy is our exclusive use of the free ALT flap for soft tissue reconstruction. Traditionally, pedicled flaps such as PM, transvers rectus abdominis muscle (TRAM), and latissimus dorsi (LD) flaps have been the go-to for fast and reliable reconstruction of large anterior chest wall defects, whereas smaller defects were closed using pedicled myocutaneous trapezius, deltoid, or scalene flaps [28]. Most authors prefer uni- or bilateral PM flaps due to their proximity, reliability, and straightforward harvest technique [4,11,13]. Representative studies on sternectomy report pectoralis major flap usage in 48.8–86% of cases [2,24]. These flaps are well-suited for defects of the anterosuperior chest wall. Preserving the thoracoacromial trunk during surgical dissection ensures optimal blood supply and a low flap loss rate (<3%) [13]. However, a significant limitation of the PM flap is its inability to cover the distal portion of the sternal region. This aspect may explain relatively higher rates of wound complications (7% wound dehiscence of the lower pole, 11% local infection leading to mesh removal) in studies with high PM flap use [7,24]. For large anterior defects or cases where the PM has been partially resected, alternative options include the latissimus dorsi, rectus abdominis or free flaps. Each of these reconstructive techniques has advantages and disadvantages (see Table 2), requiring multidisciplinary preop planning, including thoracic, plastic surgery and anesthetic teams.

In our opinion and based on our experience, free ALT flaps can safely and efficiently be used to provide soft tissue reconstruction after sternectomy. The ALT flap allows for coverage of large defects, has good, reliable blood supply, and can be harvested with minimal donor site morbidity. Additionally, operative time is reduced by both surgical teams (thoracic and plastic surgery) operating simultaneously. From a technical standpoint, our study provides novel insight into the use of the left brachiocephalic vein for venous anastomosis, which, we have found, improves blood drainage and reduces the risk of flap edema, congestion, and failure. Due to its accessible position and large caliber, the use of the left brachiocephalic vein may represent the best option for the recipient vein in free flaps following total sternectomy. In our study, surgical reintervention was necessary in the case of two patients due to flap-related complications. In one patient, ALT pedicle venous thrombosis was suspected approximately 24 h postop. Prompt detection and emergency surgery with venous anastomosis revision efficiently prevented flap loss. The other patient who required surgery for flap-related complications presented with donor site hematoma. Notably, no infections or wound dehiscence were encountered in our group. During the median 10-month follow-up period, no significant aesthetic or functional donor site impairment was recorded in the five patients included in our study. Similar results have been reported by Wise et al. in a study on 51 ALT patients [28]. However, Townley et al., in a study conducted on 97 patients who underwent free-ALT flap reconstruction, concluded that while muscle function was not impaired by intramuscular perforator dissection, reduced sensibility was frequent [29].

Our study demonstrates the feasibility and effectiveness of the “spider web” technique as a simple, cost-effective, and reliable method for stable chest wall reconstruction following extensive sternal resections. Additionally, we highlight the routinely successful use of the ALT myocutaneous flap for soft tissue reconstruction, particularly emphasizing the benefits of venous flap drainage in the left brachiocephalic vein. The results of this study support the “spider web” technique as a first-line reconstruction method for large chest wall defects, offering a viable alternative to more complex or costly techniques and the ALT flap as an optimal substitute for extensive soft tissue defects. Moreover, we propose a practical classification system for sternectomy, integrating the extent of the resection with the stability requirements of reconstruction, which may better reflect the surgical realities compared with previously published classifications.

The primary limitation of our study is the small sample size, a consequence of the rarity of this type of surgery. However, our findings align with most published studies on this topic, where the number of total sternectomies generally remains low. Further, our experience with the “spider web” technique extends beyond sternectomy, as this method has been routinely employed in our clinic for all types of chest wall reconstructions. While our results are promising, larger comparative studies are needed to establish the optimal reconstructive strategy following sternectomy and to further evaluate long-term outcomes associated with different techniques.

## 5. Conclusions

Sternectomies are rarely performed surgical procedures, primarily indicated for neoplastic pathologies and pose significant challenges for stable reconstruction. Numerous reconstruction techniques have been described, each with distinct advantages and limitations. However, establishing a standardized approach remains unfeasible due to the lack of comparative studies and the reduced number of cases included.

Based on our experience, the “spider web” technique is a simple, cost-effective, reliable method to obtain a stable chest wall reconstruction, even in extensive defects following sternectomy. In cases where the PM muscle cannot be used for soft tissue reconstruction—either due to its inability to reach the lower third of the sternum or because the resection extends laterally into the muscle—we strongly recommend the use of ALT myocutaneous flap as a large-volume, well-vascularized substitute with low local and minimal donor site morbidity. Additionally, venous anastomosis to the left brachiocephalic vein may further enhance flap viability by improving venous drainage and reducing the risk of congestion.

## Figures and Tables

**Table 1:** Overview of materials used for sternal reconstruction, their reported advantages and disadvantages, and recent developments and future perspectives [6,8,9,11,12].

Type of Method	Type of Material	Advantages	Disadvantages
Non-rigid	Braided synthetic meshes	Largely available, reduced costs, easy to handle and store, easy to adapt to defect size and configuration	
	⮚ Non-absorbable:		
	polyester mesh, polypropylene mesh;	Thin, good tissue integration can be used in combination with rigid methods (methyl methacrylate, titanium bars)	Low resistance to infection, poor protection of mediastinal organs, permeable for fluids
	Titanium mesh;	Higher rigidity and stability while preserving elasticity and chest wall dynamics, well tolerated, easy to cut and shape, good biocompatability	High costs, radiopacity, attenuation of radiotherapy
	⮚ Slowly absorbable: polyglactin mesh.	Inert, biocompatible, can be used in infected environment	Low stability, poor protection of mediastinal organs
	Compact synthetic meshes	Thicker (increased stability and mediastinal organ protection), impermeable for fluids	Limited tissue ingrowth
	Biological meshes: biological collagen matrixes derived from porcine dermis or bovine pericardium	Stable reconstruction, durability (non-absorbable), good tissue integration, allows remodeling (useful in the pediatric population), decreased infection risk	High costs, for more extensve defects coverage with muscle flap still necessary
Rigid	Methylmethacrylate: in general, used in combination with polypropylene mesh (“sandwich” technique)	Stable reconstruction even for large defects, prevents paradoxical movements and chest wall deformities	Excessive chest wall rigidity, increased pain, increased risk of infection
	Titanium bars	Stable reconstruction, biocompatible, high osseointegration, low optical density, high strength/weight ration, relative resistance to infection, easy to adapt to defect size and shape, good cosmetic results, compatible with MRI	Risk of bar fracture or displacement, higher costs
	Silicone molds injected with methylmethacrylate, systems based on rubber or Carbone fibers	Good stability, tailored reconstruction	Very limited published experience
	Autologous bone graft: iliac graft, fibula graft, rib graft	Stable reconstruction, good tissue integration	Limited size (unsuitable for large defects), increased operative time, possible donor site complications, have to be fixed with bars resulting in a “rigid plate-effect”
	Allografts (human cadaveric) and homografts (porcine):	Stable reconstruction, good tissue integration, easily adjustable to defect size and shape, no donor site complications, can be stored for long periods, reasonable costs	
	⮚ Cryopreserved ribs;		Need for tissue bank, have to be fixed with bars
	⮚ Sternal cadaveric allograft (sternal transplantation).		Limited published experience, need for tissue bank, legislatory constraints
Recent developments and perspective methods	Three-dimensional printing (titanium, polyether-ether-ketone)	Theoretical advantages: more precise adaptation to defect, better fixation systems, shorter operative time, less pain, improved aesthetics	Very limited published clinical experience
	Tissue regeneration using mesenchymal stem cells (MSCs)		

**Table 2:** Most common soft tissue reconstruction methods for covering defects after sternal resections [3,9,12,13,14,15,16,17,18].

Type of Soft Tissue Substitute	Type of Flap	Variants	Advantages	Disadvantages
Pectoralis major flap	Local advancement muscle or myocutaneous flap	Pedicled on thoracoacromial trunk	Optimal coverage of upper 1/2 of sternum defects Good blood supply (<3% failure)	Insufficient for lower 1/3 of sternum defects
	Turn-over flap (IMAP–internal mammary artery perforator flap)	Perforators from internal mammary artery	Alternative for midline defects if thoracoacromial vessels are compromised	
Rectus abdominus flaps	Pedicled myocutaneous flaps based on superior epigastric vessels	Vertical rectus abdominus myocutaneous flap (VRAM)	Can cover large defects after sternal resections	High incidence (13%) of abdominal hernias
		Transverse rectus abdominus myocutaneous flap (TRAM)	Can cover large defects of the anterolateral chest wall Poorer blood supply of the skin island as VRAM	
Latissimus dorsi flap	Pedicled muscle or myocutaneous flap	Pedicled based on the thoracodorsal vessels	Can cover large soft tissue defects Can reach every region of the anterolateral and posterior ipsilateral chest wall Can be used pedicled or as free flap	High incidence (79%) of seroma Large scar Temporary reduction in arm strength Need for patient repositioning on the operating table
Omental flap	Pedicled omentum flap	Pedicled on the right or left gastroepiploic vessels	Very useful in infected areas or reconstructions for radionecrosis Can cover large sternal defects	Requires laparatomy or laparoscopy Offers insufficient stability (combination with meshes necessary) Additional free skin graft necessary
Chimeric flap of latissimus dorsi and serratus anterior muscles	Chimeric flaps based on subscapular trunk		Good coverage of large defects	Relative high incidence of seroma due to large undermining
Anterolateral thigh (ALT) flap	Free flap based on perforators from the descending branch of the lateral circumflex femoral vessels	Can provide muscle, fascia, skin, or a combination of any of these	Relatively constant vascular supply Widely used microsurgical flap Good coverage of large defects Minimal donor site complications Two-team approach to minimize operative time possible	Require microsurgical skills Possible donor site complications (bleeding, hematoma, seroma, infection)

**Table 3:** Proposed classification by extent of sternal resections.

Type A	manubrial resection (preservation of corpus sterni)	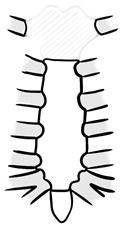
Type B	resection of corpus sterni (preservation of manubrium)	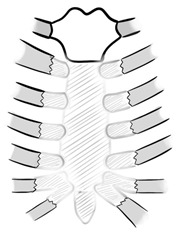
Type C	total sternal resection	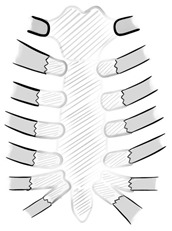

**Table 4:** Vascular anatomy classification of ALT flaps.

ALT Type	Number of Perforators	Source Vessel
I	Single	Transverse branch
II	Single	Descending branch
III	Multiple	Transverse branch
IV	Multiple	Descending branch
V	Multiple	Both branches

**Table 5:** Demographic parameters and surgery-related characteristics of the patients analyzed group.

No.	Sex	Age (Years)	Diagnosis	Medical History/Comorbidities	Tumor Size AP/CC/LL (mm)	Tumor Location and Extent	Type of Resection	Type of Reconstruction	Type of Soft Tissue Reconstruction	Surgery Duration (min)	Blood Transfusion	Postoperative LOS (Days)	Resection Margin Status	30-Day Morbidity
1.	F	57	Breast cancer metastasis	Breast cancer (invasive lobular carcinoma of the left breast, 2016) Grade 3 mitral regurgitation Grade 1 tricuspid regurgitation Osteopenia	34/70/52	Sternal body	Sternal body (type B sternal resection)	Spider-Web + polypropylene mesh	ALT flap (right side)	370	1 packed red blood cells	9	R0	No complications
2.	F	40	Recurrence of a desmoid tumor	Desmoid tumor in the left anterior chest wall, excised 2021	49/112/76	Sternal body with extension in the left anterior chest wall and left rectus abdomini muscle	Sternal body (type B sternal resection) + left anterior chest wall from 4th rib downwards and lateral to midclavicular line + superior portion of left rectus abdomini muscle up to left semilunar line	Spider-Web + polypropylene mesh	ALT flap (left side)	400	1 packed red blood cells	11	R0	Thrombosis of venous anastomosis, reintervention with desobstruction and microsurgical vascular anastomosis
3.	F	20	Hodgkin lymphoma	No	37/77/46	Large lesion originating in the sternal body and extending into manubrium sterni	Total sternectomy (type C sternal resection)	Titanium bars and polypropylene mesh	ALT flap (right side)	435	No	11	R0	No complications
4.	F	47	Breast cancer metastasis	Breast cancer (left invasive carcinoma) Sinus tachycardia Mild mitral insufficiency Silent ischemic heart disease Smoker	45/137/62	Extension into the chondrocostal cartilages of the right anterior 3rd rib, the 4th rib on both the left and right sides, the right 5th rib, and the manubrium	Total sternectomy (type C sternal resection)	Spider-Web + polypropylene mesh	ALT flap (right side)	330	No	10	R0	Pneumonia
5.	F	64	Sternal metastasis from poorly differentiated thyroid carcinoma	Poorly differentiated thyroid carcinoma pT4aNxL1V1R1 treated with total thyroidectomy and resection of the sternal manubrium, right sternoclavicular joint and first right chondrosternal joint, followed by adjuvant radiotherapy (2022) Iatrogenic hypothyroidism Class 1 obesity	4/4/2 cm	Extension into adjacent soft tissues, invades the presternalsubcutaneous tissue,	Total sternectomy (type C sternal resection)	Spider-Web + polypropylene mesh	ALT flap (right side)	390	1 packed red blood cells	14	R	Hematoma at the donor site (right thigh), surgically evacuated

## Data Availability

The data supporting the findings of this study are available from the corresponding author upon reasonable request. Due to ethical considerations, the data are not publicly accessible.
